# BDCA2 plays a central role in the binding, internalization and response of plasmacytoid dendritic cells to vidutolimod

**DOI:** 10.3389/fimmu.2026.1769287

**Published:** 2026-02-12

**Authors:** Caitlin D. Lemke-Miltner, Sue E. Blackwell, Chaobo Yin, Travis D. Fischer, George J. Weiner

**Affiliations:** 1 Holden Comprehensive Cancer Center, University of Iowa, Iowa City, IA, United States; 2 Department of Internal Medicine, University of Iowa, Iowa City, IA, United States; 3 Cancer Biology Graduate Program, University of Iowa, Iowa City, IA, United States

**Keywords:** BDCA2, *In situ* immunization, plasmacytoid dedritic cell, TLR9, virus like particle

## Abstract

**Introduction:**

Vidutolimod (Vidu) is a nanosized virus-like particle (VLP) composed of the Qβ bacteriophage capsid assembled around a Toll-like receptor 9 (TLR9) agonist. *In situ* immunization with Vidu through intratumoral injection can induce a systemic anti-tumor immune response and has shown promise in early phase cancer clinical trials. Activation of intratumoral plasmacytoid dendritic cells (pDCs), and their production of type I interferon (IFN), plays a key role in initiating the anti-tumor response to Vidu and is dependent on coating of the VLP by antibodies against the Qβ capsid (anti-Qβ). Here we evaluated the mechanisms responsible for the binding, internalization and response of pDCs to anti-Qb-coated Vidu.

**Methods:**

Using multicolor spectral flow cytometry and imaging flow cytometry, we characterized the association and uptake of anti-Qb-opsonized, fluorescently labeled Vidu by pDCs and other immune cell subsets. Interferon alpha (IFNα) secretion and differentiation marker expression on pDCs after various treatments with anti-Qβ-coated Vidu were evaluated to determine which pDC membrane proteins contribute to Vidu uptake and pDC activation.

**Results:**

Anti-Qb-coated Vidu interacted broadly with immune cells, mediated in most cases by canonical Fcγ receptors (FcγRs), i.e., CD16, CD32 and CD64. A notable exception was in the pDC population where binding was found to also be mediated by BDCA2, a type II C-type lectin. This pDC-specific receptor contributed to internalization of anti-Qβ-coated Vidu and subsequent pDC activation when lower concentrations of anti-Qb were used. In contrast, Vidu coated with higher concentrations of anti-Qb induced significant internalization of BDCA2 and reduced activation of pDCs.

**Discussion:**

These findings indicate BDCA2 plays a dual role in the immune response to Vidu, with the amount of anti-Qb antibody coating Vidu determining whether interaction of Vidu with BDCA2 results in pDC activation or inhibition. This important mechanistic information could influence the design of the next generation of pDC-targeting immunotherapeutic nanoparticles.

## Background

Immunotherapy, including immune checkpoint blockade, has had a major impact on cancer medicine. Nevertheless, many patients do not respond to such therapy. Lack of an interferon (IFN) signature within the tumor correlates with reduced rates of response (1). One approach to overcoming resistance and eliciting a stronger IFN signature is through *in situ* immunization, which utilizes direct intratumoral injection of immunostimulatory agents designed to enhance the local IFN response.

Plasmacytoid dendritic cells (pDCs) are a rare, yet crucial subset of cells that produce significant amounts of type I IFN, including IFNα, upon sensing of viral or bacterial nucleic acids via endosomal Toll-like receptors (TLR) (2–6). One such receptor is TLR9 that can be activated by synthetic CpG oligodeoxynucleotides (ODN) (7–12). *In situ* immunization with TLR9 agonists has been explored in preclinical models and clinically. Promising results from these studies demonstrate the potential of this approach to enhance the systemic anti-tumor response (13–15).

Vidutolimod (Vidu) is one such TLR9 agonist. Vidu is a virus-like particle (VLP) composed of Qβ-capsid protein encapsulating a CpG-A ODN TLR9 agonist known as G10. Vidu elicits a potent IFNα response from pDCs *in vitro* and has promising therapeutic effectiveness *in vivo* following intratumoral injection in both animal models and early phase clinical trials (16–23). The ability of Vidu to activate pDCs and induce an anti-tumor response is dependent on coating of the Vidu by antibodies specific to the VLP Qβ-capsid protein (anti-Qβ) (16). Thus, the first treatment with Vidu has little immunologic effect other than inducing production of anti-Qβ. The actual anti-tumor immune response is initiated with the second dose when the Vidu is opsonized by anti-Qβ, thereby allowing for uptake by, and activation of, pDCs.

Expression of CD32 by pDCs has been reported under some conditions (24). This led to the assumption that uptake of anti-Qβ coated Vidu by pDCs is mediated by Fcγ receptors (FcγRs) (18). However, recent evidence including from the Human Protein Atlas (www.proteinatlas.org) based on analysis of freshly isolated pDCs using more robust identification techniques indicates pDCs have minimal expression of FcγRs, i.e., CD16, CD32 and CD64 (24–26). These results suggest an additional receptor may be involved in the uptake of anti-Qβ-opsonized Vidu by pDCs.

BDCA2 is a receptor exclusively and constitutively expressed on the membrane of pDCs (27). Signaling via BDCA2 has been shown to block the IFNα response to TLR ligands (28). This has led to interest in anti-BDCA2 antibodies as potential immunosuppressive agents that inhibit pDC activation in autoimmunity (29–31). Glycosylation moieties present in the Fc region of IgG, IgA and IgM are recognized, bound and internalized by BDCA2, suggesting it can function as an alternative FcγR on pDCs (28, 32). This raises the question of whether BDCA2 might serve as a receptor for uptake of anti-Qβ coated Vidu by pDCs and contribute to its immunostimulatory effects despite its described functional role as an inhibitory receptor.

Here, we report a series of studies demonstrating that BDCA2 plays a central role in the uptake of anti-Qβ-coated Vidu by pDCs. These findings could have significant implications for our understanding of the function of BDCA2, on clinical observations to date with Vidu, and on the design of future agents for *in situ* immunization based on activation of intratumoral pDCs.

## Materials & methods

### Human samples

Peripheral blood was collected from healthy donors at the University of Iowa and used as the source of peripheral blood mononuclear cells (PBMCs) for these studies, as approved by the University of Iowa Institutional Review Board (Biomedical IRB-01). Donors were between the age of 18–75 and both sexes were represented, with no preference.

### Cell isolation and culture conditions

PBMCs were isolated from peripheral blood by standard density gradient centrifugation using Histopaque-1077 (Sigma-Aldrich) and removal of red blood cells by RBC lysis buffer (Biolegend). For culture, collected PBMCs were suspended in complete RPMI 1640 media supplemented with 10% FBS, 1X GlutaMAX, 1mM Sodium Pyruvate, 10mM HEPES (Thermo Fisher Scientific), 50µM 2-Mercaptoethanol (Sigma-Aldrich) and 50 μg/ml Gentamycin (IBI Scientific), added to 96-well U-bottom culture plates at a fixed amount (2 x 10^5^ in 200μL per well) and treated with the outlined reagents for 2 or 20 hours at 37 °C in a humidified atmosphere with 5% CO_2_.

### Vidu, anti-Qβ antibody and other reagents

Vidu and recombinant human anti-Qβ antibody were produced and provided by Checkmate Pharmaceuticals (now Regeneron). Fluorescently labeled Vidu was generated using the Invitrogen Alexa Fluor 647 Protein Labeling Kit (Thermo Fisher Scientific). Vidu (AF647 labeled or unlabeled) was used at a final protein concentration of 5 µg/mL and recombinant human anti-Qβ was used at a final concentration of 1, 5, 20 or 80 µg/ml, as noted in figure legends. Monoclonal REAfinity^®^ recombinant human antibodies targeting pDC surface antigens BDCA2 (Miltenyi, cat. #130-124-317) and BDCA4 (Miltenyi, cat. #130-124-318) were evaluated for their ability to inhibit binding and/or uptake of anti-Qβ-coated Vidu. FcγRs were blocked using polyclonal goat antibodies against human CD16 (R&D, cat. #AF1597), CD32 (R&D, cat. #AF1330) or CD64 (R&D, cat. #AF1257) (16, 33). Miltenyi REAfinity^®^ recombinant antibodies were selected because they are engineered to reduce undesirable binding to FcγRs. Goat antibodies were selected because their Fc region has low affinity for human FcγRs (34), thus limiting off-target effects. All blocking antibodies were used at a final concentration of 1 μg/ml. Species- and vendor-matched isotype control (IC) antibodies were included in blocking experiments. Prior to the addition of anti-Qβ and Vidu, antibodies against surface receptors were pre-incubated with cells for a minimum of 15 minutes and a maximum of 30 minutes at 37 °C, based on previously reported BDCA2 and CD32 blocking studies performed with PBMCs (35, 36).

### Flow cytometry

PBMC (2 x 10^5^ per well) were cultured in 96-well U-bottom plates with treatments for 2 or 20 hours. Cells were collected and stained with Zombie Violet Fixable Viability Dye (Biolegend, cat. #423114) according to manufacturer’s protocol to distinguish live from dead cells. Following this, cells were surface stained with antibodies against human CD3 (Biolegend, cat. #300336), CD4 (Biolegend, cat. #344656), CD8 (Biolegend, cat. #301038), CD11b (Biolegend, cat. #301322), CD11c (Thermo Fisher, cat. #46-0116-42), CD14 (BD, cat. #563561), CD15 (Biolegend, cat. #323033), CD16 (Biolegend, cat. #302032), CD19 (BD, cat. #749173), CD32 (Biolegend, cat. #303215), CD45 (Biolegend, cat. #368542), CD56 (Biolegend, cat. #318351), CD64 (Biolegend, cat. #305027), CD80 (BD, cat. #751732), CD123 (Biolegend, cat. #306054), BDCA2 (Biolegend, cat. #354207), BDCA4 (Biolegend, cat. #354504), HLA-DR (Biolegend, cat. #307640) and PD-L1 (Biolegend, cat. #329736). Human TruStain FcX Fc Receptor Blocking Solution (Biolegend, cat. #422302) and True-Stain Monocyte Blocker (Biolegend, cat. #426103) were included during all surface staining steps. Stained samples were acquired on a Cytek Aurora CS Spectral Flow Cytometer (University of Iowa Flow Cytometry Facility) using SpectroFlo software. Data was analyzed using FlowJo v10 software (BD Biosciences). Dead (Zombie Violet positive), CD45 negative, and aggregated cells were excluded from analysis. Positive and negative staining gates were determined with FMO controls. pDCs were identified by the following staining pattern: 1) negative for CD11b, CD11c, CD14, CD15, CD19, CD3, CD56 and 2) positive for BDCA4, CD123, and BDCA2 (when not being blocked with antibody).

### IFNα ELISA

PBMC (2 x 10^5^ per well) were cultured in 96-well U-bottom plates with treatments for 20 hours. Supernatants were collected and IFNα was measured with the VeriKine Human IFNα ELISA Kit using the extended standard range (PBL Assay Science, cat. #41100-2) according to the manufacturer’s protocol. ELISA plates were read on a ThermoMax Precision microplate reader using SoftMax^®^ microplate data acquisition software (Molecular Devices).

### Multispectral imaging flow cytometry

PBMC (2 x 10^5^ per well) were cultured in 96-well U-bottom plates with treatments for 20 hours. All samples were collected and stained with Zombie Violet Fixable Viability Dye (Biolegend, cat. #423114) according to manufacturers’ protocol to distinguish live from dead cells, followed by staining to analyze the pDC population for Vidu-AF647 uptake or internalization of BDCA2.

To analyze pDC uptake of Vidu-AF647, cells were surface stained with antibodies against human CD3 (Biolegend, cat. #300336), CD11b (Biolegend, cat. #101229), CD11c (Biolegend, cat. #337215), CD19 (Biolegend, cat. #302241), CD45 (Biolegend, cat. #368542), BDCA4 (Biolegend, cat. #354504) and BDCA2 (Biolegend, cat. #354207). Cells were left unfixed and analyzed immediately to preserve the integrity and magnitude of the Vidu-AF647 signal associated with cells.

To analyze internalization of BDCA2 in pDCs, cells were first surface stained with antibodies against human CD3 (Biolegend, cat. #300336), CD11b (Biolegend, cat. #101229), CD11c (Biolegend, cat. #337215), CD19 (Biolegend, cat. #302241), CD45 (Biolegend, cat. #368542) and BDCA4 (Biolegend, cat. #354504). This was followed by fixation and permeabilization with the FIX & PERM Cell Fixation and Cell Permeabilization Kit (Thermo Fisher Scientific, cat. #GAS003), with the staining antibody against BDCA2 (Biolegend, cat. #354207) added during the permeabilization step, as outlined in the manufacturer’s protocol.

Human TruStain FcX Fc Receptor Blocking Solution (Biolegend, cat. #422302) and True-Stain Monocyte Blocker (Biolegend, cat. #426103) were included during all surface staining steps. Samples were acquired on a Cytek Amnis ImageStream MkII Imaging Flow Cytometer (University of Iowa Flow Cytometry Facility) using INSPIRE software version ISX. Samples were run at a low flow rate with 40X or 60X magnification, as noted in figure legends, and single-color controls were collected to calculate a compensation matrix. Data was analyzed using IDEAS software version 6.2 (Cytek). Events that were out of focus, aggregated, CD45 negative or dead (Zombie Violet positive) were excluded from analysis. pDCs were identified as: 1) negative for CD11b, CD11c, CD3 and CD19 and 2) positive for BDCA2 and/or BDCA4. Internalized Vidu-AF647 signal was quantified with the IDEAS software using an internalization mask defined by 75% erosion from the brightfield (BF) cell membrane outline to stringently detect particle uptake and exclude background noise. Internalized BDCA2 was quantified with the IDEAS software using the Internalization Wizard with “Cell Image” defined by CD45 and the “Internalizing Probe” defined as BDCA2.

### Statistical analysis

Statistical analyses were performed in GraphPad Prism (v10.2.2). Data are presented as a mean with individual donor/cell data points shown. Paired sample t-tests were used to compare two groups with matched donors. Repeated measures one-way ANOVA with Dunnett’s multiple-comparisons test or two-way ANOVA with Sidak’s multiple comparisons test were used for comparisons of more than two groups, as noted in figure legends. Data were significant when p values ≤ 0.05 (*, p ≤ 0.05; **, p ≤ 0.01; ***, p ≤ 0.001; ****, p ≤ 0.0001). Data were not significant (ns) when p values > 0.05.

## Results

### Anti-Qβ promotes Vidu association with all immune cell populations

**Figure 1:**
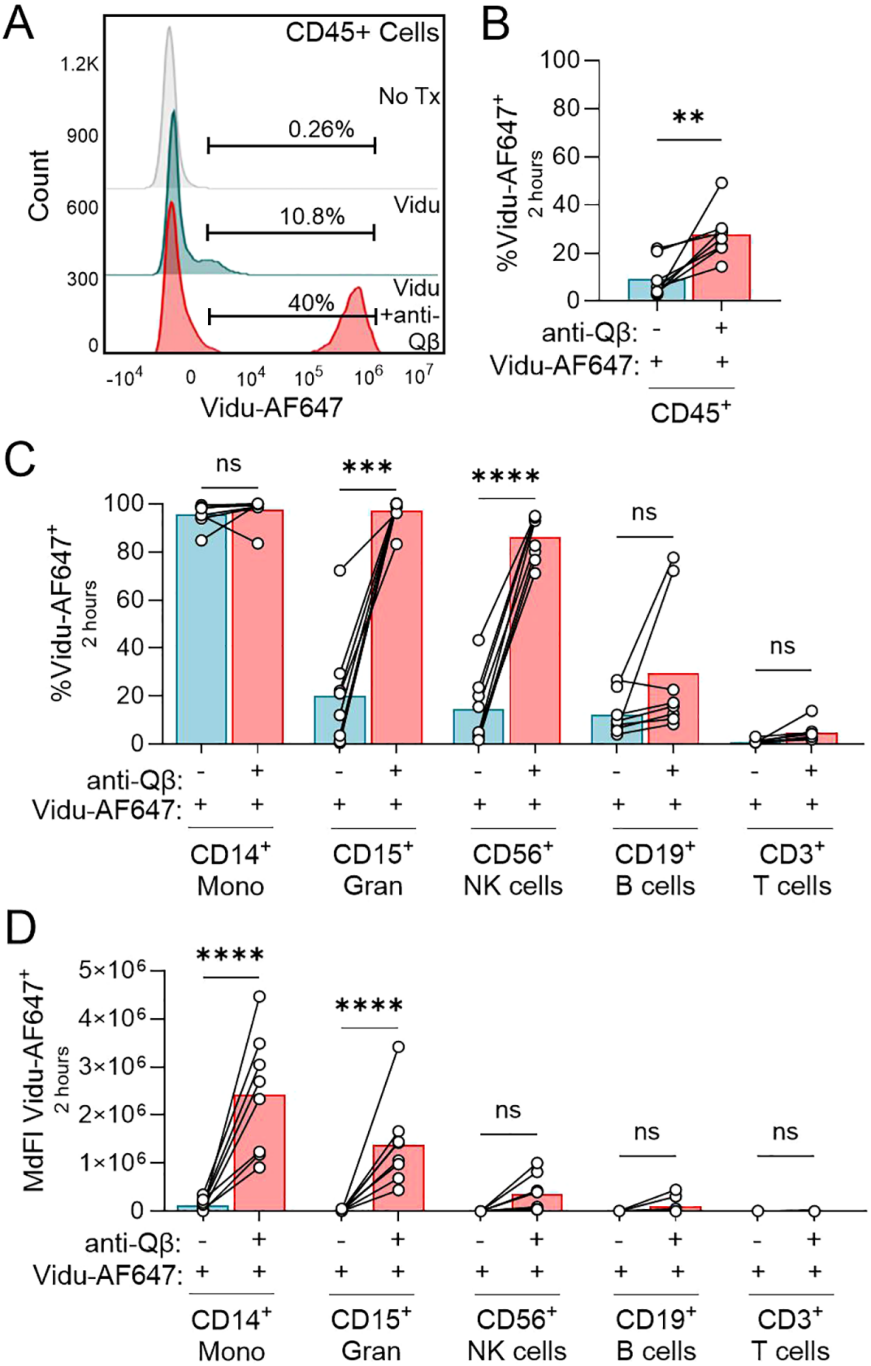
Anti-Qβ enhances the interaction of Vidu with multiple immune cell populations. **(A-D)** PBMCs from healthy donors (n=8) were cultured for 2 hours in media (No Tx), with Vidu-AF647 alone or with Vidu-AF647 and anti-Qβ. Vidu-AF647 signal associated with immune cell populations was determined by flow cytometry. **(A)** Gating on Vidu-AF647^+^ CD45^+^ cells from one representative donor after culturing PBMCs without treatment (grey histogram), with Vidu-AF647 (blue histogram) or with Vidu-AF647 and anti-Qβ (red histogram). **(B)** Frequency analysis of Vidu-AF647^+^ CD45^+^ cells detected by flow cytometry. **(C)** Frequency analysis and **(D)** MdFI of Vidu-AF647 signal associated with various immune cell populations. Anti-Qβ and Vidu-AF647 were each used at a final concentration of 5 μg/ml. Statistical significance was determined using a paired t-test **(B)** or two-way ANOVA with Sidak’s multiple comparisons test **(C, D)**: **p<0.01, ***p<0.001, ****p<0.0001, ns, not significant.

We previously reported that anti-Qβ is required for Vidu to activate pDCs and induce an immune response in both murine and human models (16). To further evaluate how anti-Qβ mediates this response, we used flow cytometry to assess how anti-Qβ impacts on the association of various human immune cells with Vidu. The term “association” is used for these initial results because flow cytometry is not able to distinguish between adherence of Vidu to the surface of cells and uptake of Vidu by cells. Fluorescently tagged Vidu (Vidu-AF647) was added to human PBMCs, with or without recombinant human IgG anti-Qβ, and the association of the Vidu-AF647 with various immune cell types determined by flow cytometry (**Figure 1**). After a 2-hour culture, all CD45^+^ cell subsets exhibited a relatively low level of detectable Vidu-AF647 association in the absence of anti-Qβ that significantly increased in the presence of anti-Qβ (**Figures 1A, B**). Association of Vidu-AF647 as demonstrated by percent positivity was most pronounced with monocytes (CD14^+^ Mono) while association of Vidu with T cells (CD3^+^) was limited (**Figure 1C**). Anti-Qβ co-incubation with Vidu significantly increased the frequency of Vidu-AF647^+^ granulocytes (CD15^+^ Gran) and NK cells (CD56^+^), with B cells (CD19^+^) from some donors also exhibiting an increase (**Figure 1C**). Median fluorescent intensity (MdFI) of Vidu-AF647 signal was most significantly affected by the addition of anti-Qβ in the monocyte and granulocyte cell population (**Figure 1D**). Incubation for 2 hours at 4 °C was assessed to determine the impact of internalization on anti-Qβ-coated Vidu-AF647 signal (**Supplementary Figure 1**). Vidu-AF647 signal was similar at 4 °C and 37 °C, demonstrating minimal effect of internalization on overall VLP binding. Thus, the low level of Vidu-AF647 signal associated with monocytes in the absence of anti-Qβ (**Figures 1C, D**) likely reflects surface-binding. These findings are consistent with prior research showing that monocytes can interact directly with bacteriophages (37).

### Expression of CD32 and BDCA2 by various cell types

Given the inconsistent data regarding FcγR expression on human pDCs, we characterized surface expression levels of CD32 and BDCA2 across immune cell types from multiple healthy donors by flow cytometry. As expected, CD32 was highly expressed on monocytes, granulocytes and B cells, and was present at very low levels on NK cells, T cells and pDCs (**Supplementary Figure 2A**). In contrast, surface BDCA2 expression at significant levels was limited to pDCs (**Supplementary Figure 2B**).

### Vidu association with, and activation of, pDCs requires anti-Qβ and is mediated by CD32 and BDCA2

**Figure 2:**
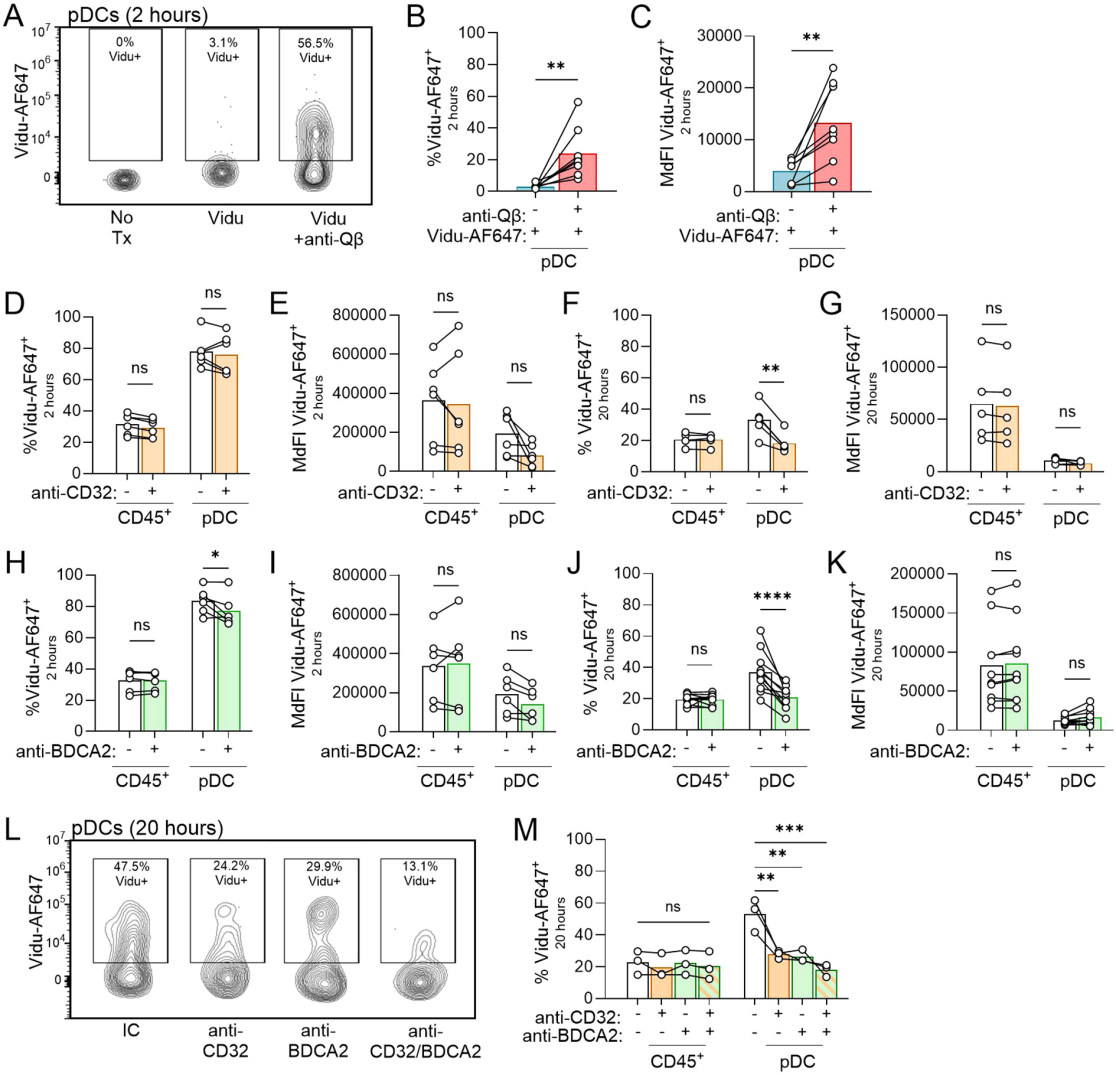
The interaction of Vidu-AF647 with pDCs, as determined by flow cytometry, is dependent on anti-Qβ and is mediated through both CD32 and BDCA2. **(A-C)** PBMCs from healthy donors were cultured for 2 hours in medium (No Tx), Vidu-AF647 alone or Vidu-AF647 and anti-Qβ. **(D-M)** PBMCs from healthy donors were cultured for 2 hours or 20 hours with Vidu-AF647 and anti-Qβ with or without receptor blocking. IC or receptor-specific antibodies (anti-CD32 or anti-BDCA2) were added to PBMC cultures prior to Vidu-AF647 and anti-Qβ. Vidu-AF647 signal associated with CD45^+^ cells or BDCA4^+^ pDCs was determined by flow cytometry. **(A)** Gating on Vidu-AF647^+^ pDCs in PBMCs from one representative donor. **(B)** Frequency and **(C)** MdFI of Vidu-AF647^+^ pDCs (n=9 donors). **(D)** Frequency and **(E)** MdFI of Vidu-AF647^+^ CD45^+^ or pDCs after PBMC from healthy donors (n=6) were treated with IC (–) or anti-CD32 (+) followed by a 2-hour culture with anti-Qβ and Vidu-AF647. **(F)** Frequency and **(G)** MdFI of Vidu-AF647^+^ CD45^+^ cells or pDCs after PBMC from healthy donors (n=5) were treated with IC (–) or anti-CD32 (+) followed by a 20-hour culture with anti-Qβ and Vidu-AF647. **(H)** Frequency and **(I)** MdFI of Vidu-AF647^+^ CD45^+^ cells or pDCs after PBMC from healthy donors (n=6) were treated with IC (–) or anti-BDCA2 (+) followed by a 2-hour culture with anti-Qβ and Vidu-AF647. **(J)** Frequency and **(K)** MdFI of Vidu-AF647^+^ CD45^+^ cells or pDCs after PBMC from healthy donors (n=12) were treated with IC (–) or anti-BDCA2 (+) followed by a 2-hour culture with anti-Qβ and Vidu-AF647. **(L)** Contour plots from one representative donor showing Vidu-AF647^+^ pDCs and **(M)** frequency of Vidu-AF647^+^ CD45^+^ or pDCs (n= 3 donors) after IC, anti-CD32, anti-BDCA2 or anti-CD32/BDCA2 treatment of PBMCs followed by a 20-hour culture with Vidu-AF647 and anti-Qβ. Antibody pre-treatment was done at a final concentration of 1 μg/ml for 15–30 minutes; anti-Qβ and Vidu-AF647 were each used at a final concentration of 5 μg/ml. Statistical significance was determined using a paired t-test **(B, C)** or a two-way ANOVA with Sidak’s multiple comparisons test **(D-K, M)**: *p<0.05, **p<0.01, ***p<0.001, ****p<0.0001, ns, not significant.

Given the vital role pDCs play in the response to Vidu, particular focus was paid to the impact of anti-Qβ on the association of Vidu with pDCs. Anti-Qβ markedly enhanced association of Vidu with pDCs, in terms of both frequency (%) and magnitude (MdFI) across multiple healthy donors after a 2-hour incubation (**Figures 2A-C**). Anti-CD32 induced little change in the frequency of anti-Qβ-coated Vidu-AF647^+^ pDCs (~2% reduction in positive cells, **Figure 2D**), although there was a trend towards anti-CD32 reducing Vidu-AF647 signal per pDC indicated by MdFI (**Figure 2E**). After a longer incubation (20 hours), anti-CD32 induced a more consistent and significant drop in the frequency of Vidu-AF647^+^ pDCs (~15% reduction, **Figure 2F**), but did not have a significant impact on Vidu-AF647^+^ pDCs MdFI (**Figure 2G**). One possible explanation for the difference in magnitude of effect between 2 hours and 20 hours is that CD32^hi^ cells (non-pDCs) are present in greater numbers and are more rapidly saturated with antibody, while CD32^lo^ pDCs present in a lower number are more slowly saturated and blocked (**Supplementary Figure 2A**). Antibodies to CD16 (FcγRIII) or CD64 (FcγRI) had no clear effect on the interactions between anti-Qβ-coated Vidu-AF647 and pDCs (0% reduction, **Supplementary Figures 3A, B**). Anti-CD16, but not anti-CD64, reduced the magnitude (MdFI) of anti-Qβ-coated Vidu association with other CD45^+^ cell types (**Supplementary Figures 3C-E**).

Given that antibody to CD32 did not fully inhibit binding of anti-Qβ-coated Vidu to pDCs, we evaluated whether other receptors were involved. The pDC-specific receptor BDCA2 was of particular interest because it is known to bind to IgG via glycosylation moieties found in the constant region (28). Blocking BDCA2 had a time-dependent effect on anti-Qβ-coated Vidu association with pDCs. Anti-BDCA2 had a modest effect on the fraction of pDCs associated with anti-Qβ-coated Vidu association at 2 hours (~6% reduction). This impact increased to 16% by 20 hours. The magnitude (MdFI) of anti-Qβ-coated Vidu uptake by those pDCs that were positive was not significantly altered by anti-BDCA2 (**Figures 2H–K**). This suggests that early interactions of anti-Qβ-coated Vidu with pDCs are relatively BDCA2-independent, whereas the receptor significantly influences cumulative binding and internalization over time. The combination of both anti-CD32 and anti-BDCA2 reduced interaction between anti-Qβ-coated Vidu and pDCs to a greater degree than either antibody alone, further supporting a role for both receptors in binding of anti-Qβ-coated Vidu to pDCs (**Figures 2L,M**). We also tested blocking with an antibody against an unrelated pDC-specific receptor, BDCA4, and found anti-BDCA4 had no significant impact on the anti-Qβ-coated Vidu interaction with pDCs (**Supplementary Figures 3F, G**).

**Figure 3:**
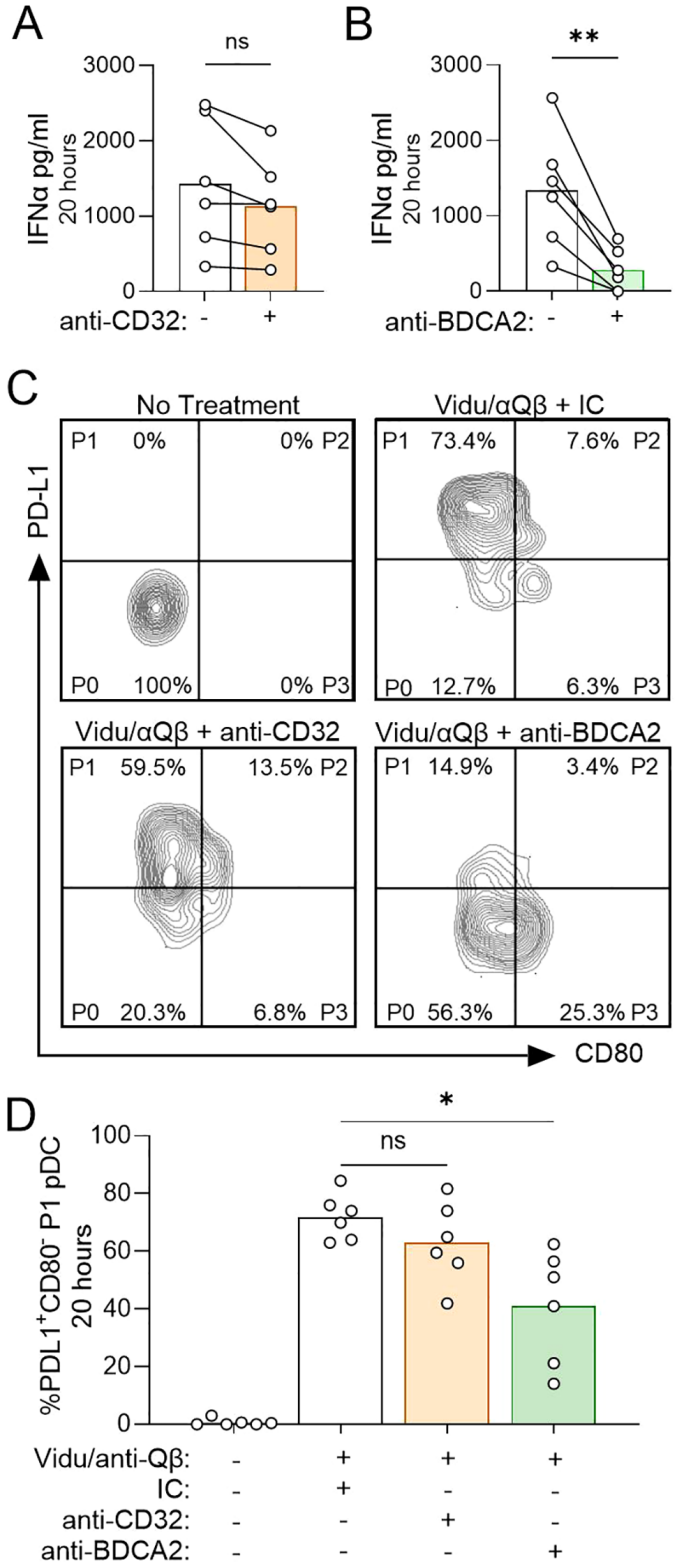
Anti-BDCA2 blocks anti-Qβ-coated Vidu induced pDC activation and differentiation. **(A, B)** IFNα levels detected by ELISA in culture supernatants obtained 20 hours after PBMC from healthy donors (n=6) were treated with IC (white bars), anti-CD32 (orange bar) or anti-BDCA2 (green bar) prior to addition of anti-Qβ and Vidu-AF647. **(C)** Flow cytometry contour plots from one representative donor and **(D)** frequency of PDL1^+^CD80^-^ P1 pDC detected across multiple donors (n=6) showing the impact of IC, anti-CD32 or anti-BDCA2 treatment on the expression of PD-L1 and CD80 on BDCA4^+^ pDCs after PBMC were cultured for 20 hours with anti-Qβ and Vidu-AF647 (subsets of pDCs are referred to as P0, P1, P2 or P3); PBMC cultured alone were stained to show background expression levels (No Treatment). Antibody pre-treatment was done at a final concentration of 1 μg/ml for 15–30 minutes; anti-Qβ and Vidu-AF647 were used at final concentrations of 5 μg/ml for 20 hours. Statistical significance was determined using a paired t-test **(A, B)** or one-way ANOVA with Dunnett’s multiple-comparisons test **(D)**: *p<0.05, **p<0.01, ns, not significant.

We then evaluated the functional impact of blocking CD32 and BDCA2 on anti-Qβ and Vidu activation of pDCs. Because we are using Vidu labeled with AF647, we confirmed that the fluorescent label did not alter the IFNα response from pDCs when treated with anti-Qβ-coated Vidu (**Supplementary Figure 4**). After a 20-hour culture, anti-BDCA2 was more effective than anti-CD32 at blunting IFNα production in response to anti-Qβ-opsonized Vidu-AF647 (**Figures 3A, B**). Next, we characterized surface expression of PD-L1 and CD80 on pDCs to determine how each blocking antibody affected pDC differentiation into previously defined subsets (38). Anti-BDCA2 was consistently more effective than anti-CD32 at blunting pDC differentiation from the immature P0 subset (PDL1^-^CD80^-^) to the type I IFN-producing P1 (PDL1^+^CD80^-^) subset (**Figures 3C, D**).

### BDCA2 is important for internalization of anti-Qβ-opsonized Vidu by pDCs

**Figure 4:**
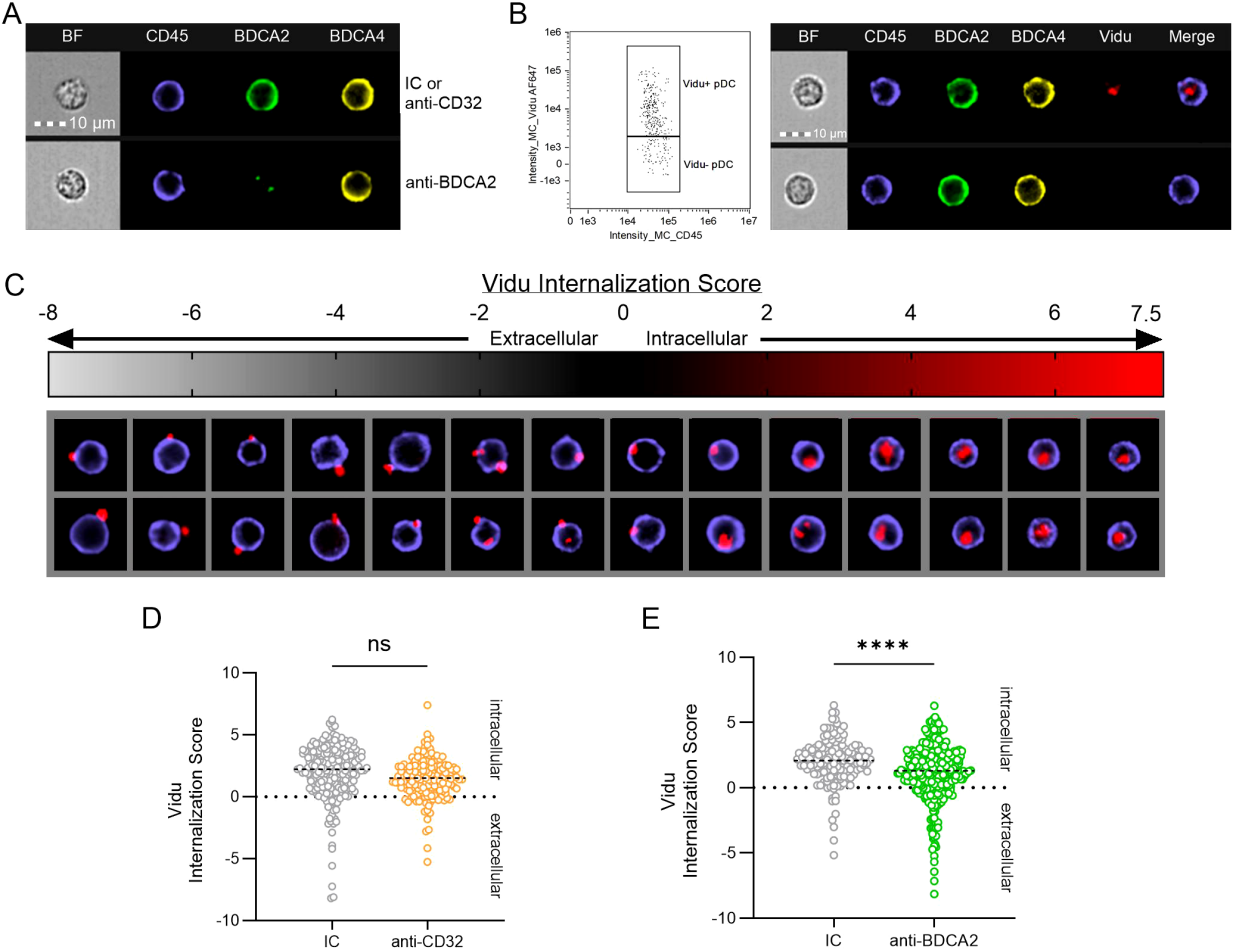
Antibody against BDCA2 reduces internalization of anti-Qβ-coated Vidu-AF647 by pDCs, as determined by multicolor imaging flow cytometry. **(A-E)** PBMCs from healthy donors were cultured for 20 hours with Vidu-AF647 and anti-Qβ. IC, anti-CD32 or anti-BDCA2 antibodies were added to PBMC cultures prior to Vidu-AF647 and anti-Qβ. Localization of Vidu-AF647 signal associated with pDCs was visualized and analyzed with IDEAS software. **(A)** Representative images of pDCs from healthy donors. Individual pDC morphology (BF), surface staining of CD45 (purple), BDCA2 (green) and BDCA4 (yellow) are shown for samples treated with anti-CD32 (or IC) or anti-BDCA2. **(B)** Representative gating and images of Vidu-AF647^+^ and Vidu-AF647^-^ pDCs; ‘Merge’ image includes CD45 and Vidu (red) signal. **(C)** Representative images of Vidu-AF647^+^ pDCs reflecting the range of Vidu Internalization Scores calculated with IDEAS software. **(D, E)** Vidu-AF647 Internalization Scores signal from individual pDCs collected from matched donors (n=4) treated with IC, anti-CD32 or anti-BDCA2 prior to culture with anti-Qβ and Vidu-AF647. Antibody pre-treatment was done at a final concentration of 1 μg/ml for 15–30 minutes; anti-Qβ and Vidu-AF647 were each used at a final concentration of 5 μg/ml. Samples were acquired at 40X magnification on Amnis ImageStream MkII. Statistical significance was determined using a paired t-test: ****p<0.0001, ns, not significant.

To determine if treating cells with receptor-blocking antibody might also affect internalization of Vidu, cellular location (intracellular versus extracellular) within the pDC population was assessed using multispectral imaging flow cytometry. PBMCs (n=4 matched donors) were treated with IC, anti-CD32 or anti-BDCA2 prior to adding anti-Qβ and Vidu-AF647 for 20hrs. Vidu^+^ pDCs were identified by a specific gating strategy (**Supplementary Figure 5**), visualized to see morphology (brightfield-BF), surface expression of CD45, BDCA2 and BDCA4 (**Figure 4A**) and gated based on Vidu signal (**Figure 4B**). Vidu-AF647^+^ pDCs were analyzed using the IDEAS software to calculate internalization scores (ranging from -8 to 7.5) which correspond to how much of the Vidu signal is extracellular versus intracellular within each cell (**Figure 4C**). While both anti-CD32 and anti-BDCA2 influenced internalization, anti-BDCA2 was more effective and significant (mean internalization = 1.117, 45% reduction) than anti-CD32 (mean internalization = 1.454, 27% reduction) at reducing pDC internalization of anti-Qβ-coated Vidu when compared to their species-matched IC (mean internalization = ~2) (**Figures 4D, E**). Further assessment of the cumulative effect of combining blocking antibodies are needed and planned for future mechanistic studies.

### Vidu coated with higher concentrations of anti-Qβ induces BDCA2 internalization

**Figure 5:**
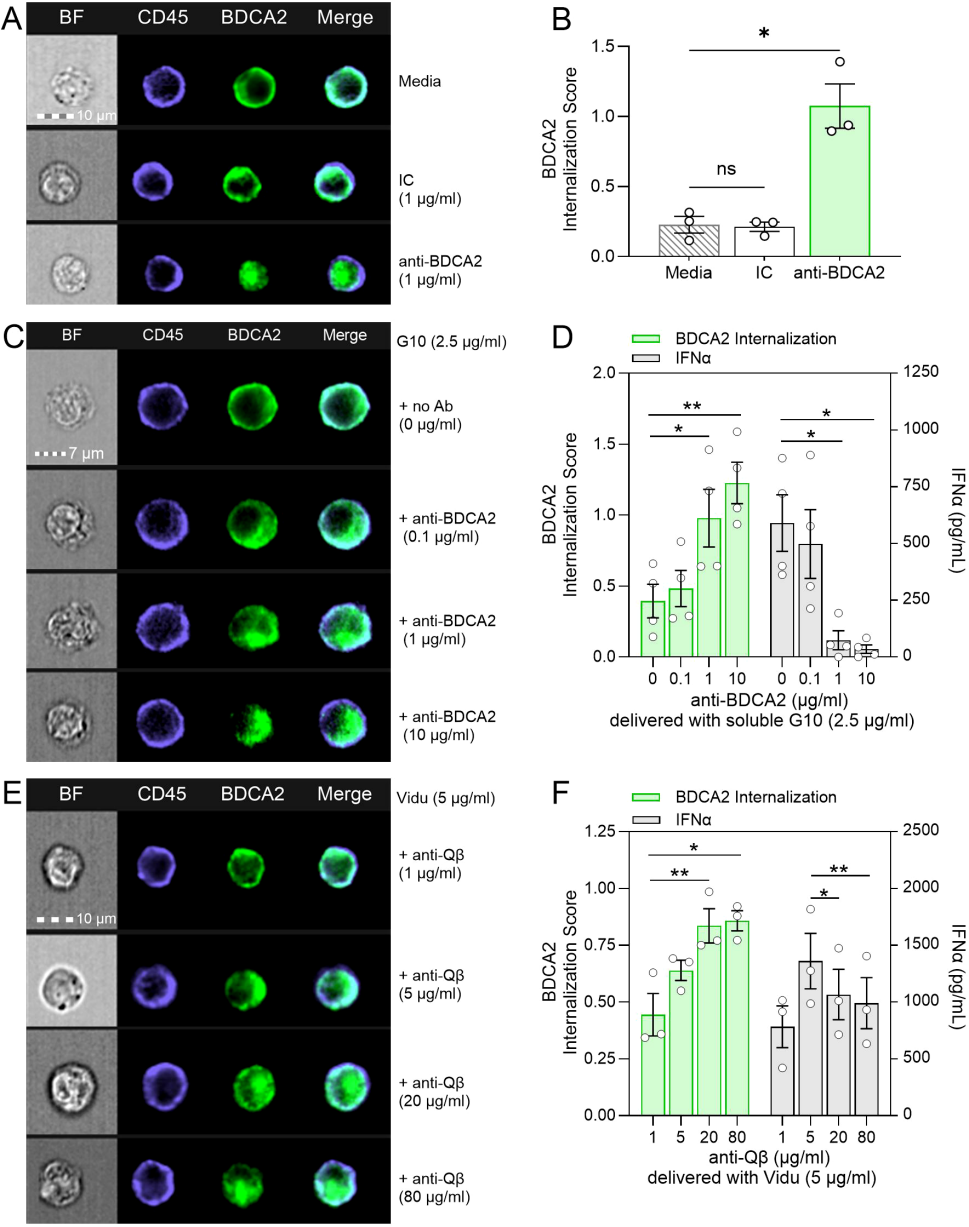
Anti-Qβ dose impacts on Vidu-induced BDCA2 internalization and on the IFNα response to TLR9 stimulation. **(A-F)** PBMCs from healthy donors were cultured for 20 hours with IC, anti-BDCA2, G10 and anti-BDCA2, or Vidu and anti-Qβ. Localization of the BDCA2 signal in pDCs was detected by both surface and intracellular staining and visualized by multicolor imaging flow cytometry. BDCA2 Internalization Scores were calculated using IDEAS software; cells shown represent the average score for the treatment. IFNα was measured by ELISA in cell culture supernatants. **(A)** Representative images of CD45^+^ (purple) BDCA2^+^ (green) pDCs left untreated (Media), treated with IC or anti-BDCA2 (1 μg/ml). **(B)** Average BDCA2 Internalization Scores calculated from samples (3 donors) treated as described in **(A)**. **(C)** Representative images of CD45^+^ BDCA2^+^ pDCs treated with G10 CpG-A (2.5 μg/ml) and varying doses of anti-BDCA2. **(D)** Average BDCA2 Internalization Scores (left y-axis, green bars) and IFNα levels (right y-axis, grey bars) from samples (4 donors) treated as described in **(C)**. **(E)** Representative images of CD45^+^ BDCA2^+^ pDCs after being cultured with a fixed amount of Vidu and varying concentrations of anti-Qβ. **(F)** Average BDCA2 Internalization Scores (left y-axis, green bars) and IFNα levels (right y-axis, grey bars) from PBMC (3 donors) treated as described in **(E)**. G10 was used at a final concentration of 2.5 μg/ml and Vidu was used at a final concentration of 5 μg/ml. Samples were acquired at 60X magnification on Amnis ImageStream MkII. Statistical significance was determined using a one-way ANOVA with a Dunnett’s multiple comparison test: *p<0.05, **p<0.01, ns, not significant.

BDCA2 crosslinking is known to induce BDCA2 internalization. This then results in a signaling cascade that blocks the pDC type I IFN response to TLR stimulation (27, 35, 39). Consistent with these reports, multispectral imaging flow cytometry demonstrated anti-BDCA2 antibody induces significant BDCA2 internalization by pDCs compared to media or IC (**Figures 5A, B**). The published literature suggests BDCA2 is an inhibitory receptor and that BDCA2 engagement and internalization impedes the ability of pDCs to respond to TLR9 stimulation (27, 35, 39–41). We therefore tracked the level of BDCA2 internalization and IFNα production by pDCs in response to different doses of anti-BDCA2 delivered with G10 (a soluble CpG-A ODN TLR9 agonist). BDCA2 internalization was not observed to any significant degree after treatment with G10 alone but did visibly increase in a dose-dependent manner as the anti-BDCA2 concentration increased (**Figure 5C**). Significantly higher internalization of BDCA2 was seen across 4 donors at higher anti-BDCA2 antibody concentrations (**Figure 5D**). BDCA2 internalization correlated with a decrease in IFNα levels detected in cell culture supernatants after 20 hours of stimulation with G10 (**Figure 5D**). These results confirm a link between BDCA2 internalization and inhibition of the IFNα response to a TLR9 agonist.

**Figure 6:**
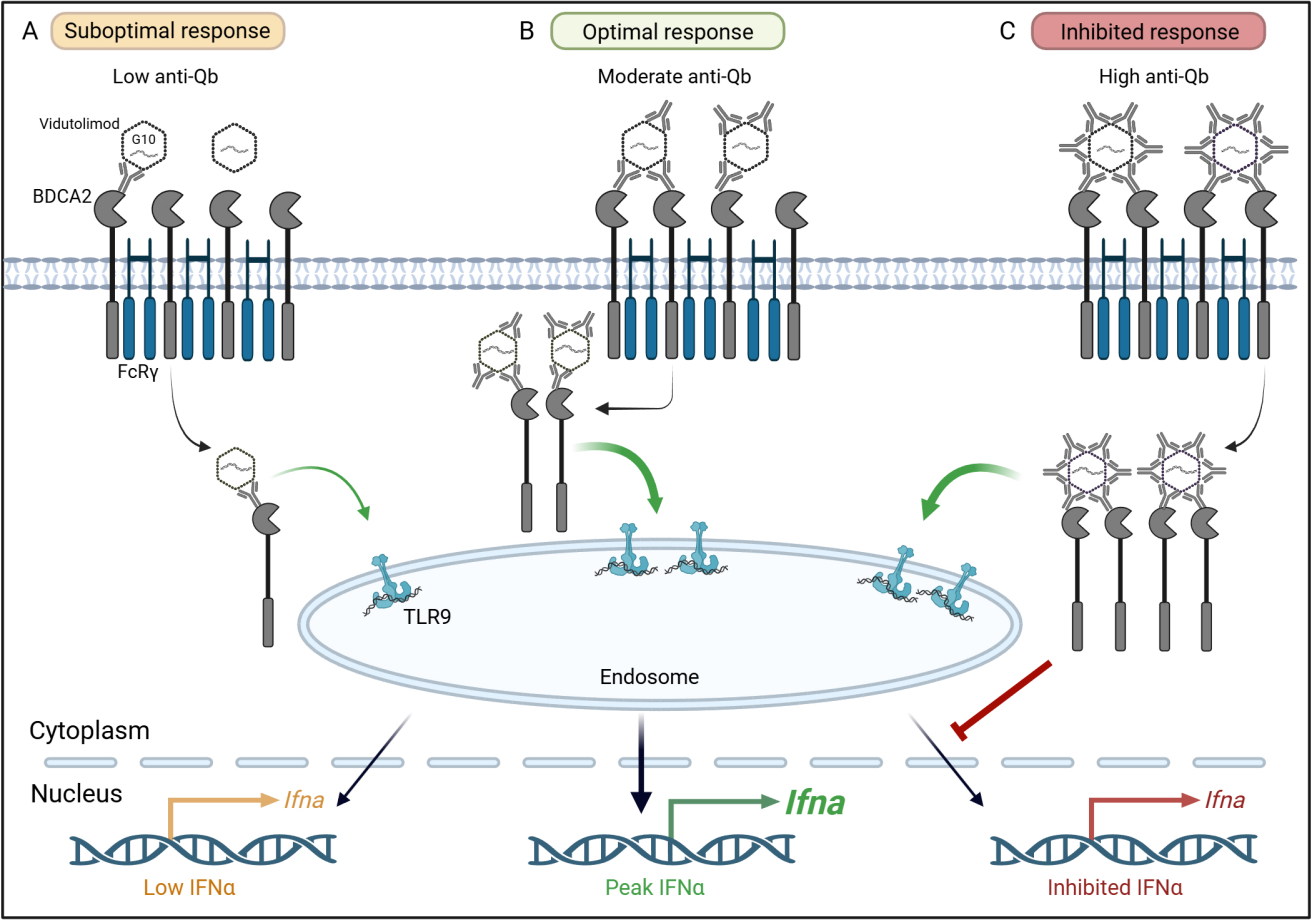
The “Goldilocks Effect” of anti-Qβ concentration, BDCA2 internalization and the IFNα response to Vidu. **(A)** Suboptimal response: At low anti-Qβ concentrations, minimal Vidu uptake occurs, resulting in weak TLR9 pathway activation and low IFNα production. **(B)** Optimal response: Moderate anti-Qβ concentrations facilitate peak IFNα production by maximizing Vidu uptake while maintaining low levels of BDCA2 internalization. **(C)** Inhibitory response: High anti-Qβ concentrations induce significant BDCA2 internalization following Vidu uptake, which suppresses the TLR9-mediated IFNα response.

Given the data indicating a role for BDCA2 as a receptor for anti-Qβ-coated Vidu and that this results in activation (not suppression) of an IFNα response, we evaluated this apparent paradox further by testing how anti-Qβ-coated Vidu affected BDCA2 internalization in pDCs. PBMCs were incubated with a constant amount of Vidu and increasing concentrations of anti-Qβ for 20 hours prior to analysis of BDCA2 internalization on pDCs. In pilot studies, signal from labeled Vidu (Vidu-AF647) was diminished after the fixation/permeabilization needed to detect intracellular BDCA2, thus unlabeled Vidu was used for these experiments. We previously reported that Vidu delivered alone (0 μg/ml anti-Qβ) does not induce an IFNα response (16, 42). Thus, we began testing the effect of anti-Qβ at a sub-optimal level (1 μg/ml) based on IFNα dose-response curves we have previously tested (42). The degree of BDCA2 internalization steadily increased as anti-Qβ levels increased from 1 μg/ml to 80 μg/ml, with the highest levels of internalization occurring at 20 μg/ml and 80 μg/ml (**Figures 5E, F**). Consistent with our prior observations, 1 μg/ml anti-Qβ delivered with Vidu stimulated minimal IFNα response and also low BDCA2 internalization. The IFNα response peaked at an anti-Qβ concentration of 5 μg/ml, when BDCA2 internalization was modest. At higher anti-Qβ concentrations (20 μg/ml to 80 μg/ml), there was a stepwise decrease in the IFNα response. These higher doses of anti-Qβ were those that induced a greater degree of BDCA2 internalization. It is important to note that even at 20-80 μg/ml of anti-Qβ, the degree of BDCA2 internalization did not reach that seen with 1-10 μg/ml of anti-BDCA2 which can explain why the IFNα response was not fully suppressed even at high anti-Qβ concentrations. These data suggest that the amount of anti-Qβ coating Vidu is a critical determinant of the IFNα response. Thus, there appears to be a “Goldilocks Effect” to explain the “bell-shaped” relationship observed between anti-Qβ concentration, BDCA2 internalization and the IFNα response (**Figure 6**). If anti-Qβ is too low, then optimal uptake of Vidu and stimulation of pDCs by the TLR9 agonist is not achieved (**Figure 6A**). When anti-Qβ is too high, TLR9 agonist is delivered, but BDCA2 crosslinking inhibits TLR9 activation (**Figure 6C**). It is only at intermediate concentrations of anti-Qβ that there is adequate uptake of the TLR9 agonist without crosslinking and downstream inhibitory signaling mediated by BDCA2 (**Figure 6B**).

## Discussion

*In situ* immunization is not a new concept. Indeed, it goes back to the late 1800s when Dr. William Coley injected bacteria into tumors and observed anti-tumor immune responses in some of his patients. Dr. Coley had no understanding of mechanism, his studies were uncontrolled, and his results were difficult to reproduce. Nevertheless, this early experience provided anecdotal evidence that cancer immunotherapy could be effective and provided the impetus for continued exploration of the biology behind the observed clinical responses (43, 44). Progress was slow for nearly a century until our understanding of the immune system caught up with our desire to leverage it to treat cancer. Since the 1980s, our understanding of immunostimulatory pathways such as TLR and T cell activation has led to the development of new agents such as Vidu with promising clinical results. Nevertheless, these agents are far from ideal, and there is still much to learn about the mechanisms of action and resistance to therapy based on such agents.

One area where we have made significant progress is our understanding of dendritic cell biology (2, 45). We have learned that a rare but important subset of dendritic cells, pDCs, are central to initiating the local immune response including an anti-tumor response within the tumor microenvironment (TME) (46, 47). When activated, pDCs produce large amounts of type I interferon locally. This type I interferon, along with other signals, activates a variety of other cell types and eventually leads to an anti-tumor T cell response (33, 42).

We previously reported that Vidu on its own does not activate pDCs. However, Vidu is highly effective at activating pDCs after it is coated by antibody against its capsid, the Qβ protein (16, 33). Here, we extend our understanding of how anti-Qβ-coated Vidu activates pDCs. More specifically, we demonstrate that the pDC receptor BDCA2 plays a central role in the ability of antibody-coated Vidu to activate pDCs.

BDCA2 is a C-type lectin selectively expressed by pDCs (40, 48). BDCA2 has previously been recognized as an inhibitory receptor, as signaling via BDCA2, such as through cross-linking via anti-BDCA2 antibodies, significantly downregulates the ability of pDCs to produce type I IFN in response to TLR7 or TLR9 agonists (27, 29, 35, 39, 49). This has led to the development of antibodies against BDCA2 as therapeutics for autoimmunity (30, 31, 35, 50).

In contrast to prior publications that focus on the suppressive effects of BDCA2 on pDC activation, we find that under select conditions, BDCA2 plays a key role in the ability of anti-Qβ-coated Vidu, with its TLR9 agonist payload, to activate pDCs. A variety of glycan ligands, including those present in the constant region of IgG, bind to BDCA2 (28, 32). Here, we report that the uptake of Vidu by multiple subsets of PBMCs is enhanced by anti-Qβ antibody. We found this uptake is mediated by traditional FcγRs (CD16, CD32, CD64) for most cell types. A notable exception is pDCs where uptake is mediated largely by BDCA2. Blocking BDCA2 reduces pDC internalization of anti-Qβ-coated Vidu, pDC production of IFNα and pDC differentiation. This effect is dependent on anti-Qβ level. Lower concentrations of anti-Qβ antibody enhance the ability of Vidu to induce IFNα production, while higher concentrations of anti-Qβ induce greater internalization of BDCA2 and reduce pDC activation and IFNα production. We are currently evaluating the impact of anti-Qβ-coated Vidu on inhibitory signaling pathways downstream of BDCA2 crosslinking, i.e. Syk recruitment and phosphorylation, calcium mobilization and reduced IRF7 translocation to the nucleus (49, 51, 52) to better understand the “Goldilocks Effect” on activation of pDCs by anti-Qβ-coated Vidu (**Figure 6**), as well as by other BDCA2-targeting ligands of interest.

This dual role of BDCA2 could help explain the observed patterns of clinical immunologic and therapeutic response to Vidu. Clinically, patients have no evidence of an immunologic or clinical response when anti-Qβ is absent from the serum, i.e., at the time of the first dose of Vidu. This first dose serves to induce production of anti-Qβ antibody. Responses suggesting immune activation are seen in patients (and in mice) with the second dose, i.e., after anti-Qβ antibody is generated. Patients treated with multiple doses of Vidu develop very high levels of anti-Qβ antibody, often exceeding 1.4 mg/ml (unpublished observation). Clinical evidence of immune activation tends to peak after 2–3 doses of Vidu, then wanes as Vidu therapy proceeds (18). Surpassing the optimal level of anti-Qβ antibody may explain the development of immune, and potentially therapeutic resistance because high levels of anti-Qβ coating Vidu induces internalization of BDCA2 in the pDC population and provides an inhibitory signal that blocks the production of IFNα.

These studies were done *in vitro* using PBMCs from healthy donors as a surrogate for intratumoral immune cells. While we observed consistent trends across our experiments, there was inherent inter-donor variability in the degree of anti-Qβ-coated Vidu interaction with pDCs, the magnitude of pDC activation, and the precise concentration of anti-Qβ antibody required to reach the opsonization “sweet spot”. Such variability likely includes individual differences in pDC frequency, the activation status of freshly isolated PBMCs, and baseline BDCA2 or CD32 expression levels, all of which could influence the threshold for BDCA2-mediated uptake and inhibition versus activation. Clearly the TME is different from the peripheral blood based on multiple factors including the activation status of cells present in the tumor, stromal cells, proteins in the extravascular tumor fluid and metabolic differences such as oxygen concentration. Further, intratumoral pDCs are phenotypically and functionally different than circulating pDCs since they have been shown to express a more immunosuppressive phenotype in multiple cancer types, which is influenced in part by tumor-associated ligands interacting with inhibitory receptors, such as BDCA2 (53–57). We strongly considered exploring the importance of BDCA2 in the uptake of anti-Qβ-coated Vidu by pDCs, and subsequent activation of those pDCs, *in vivo*. However, as presented above, anti-BDCA2 antibody turns off the response of pDCs to TLR stimulation in a manner that is independent of the role of BDCA2 in the uptake of Vidu by pDCs. The inhibitory impact of signaling via BDCA2 has led to the ongoing clinical development of anti-BDCA2 antibodies for the treatment of autoimmunity, including lupus (31). Thus*, in vivo* studies demonstrating BDCA2 blockade modulates the anti-tumor effect of Vidu would confirm what is known about BDCA2 but would not address the hypothesis that is the focus of this research, which is that BDCA2 plays a central role in efficacy of Vidu by facilitating the uptake of anti-Qβ-coated Vidu by pDCs. An additional complicating factor for doing *in vivo* studies is lack of clarity in the mouse homolog of human BDCA2. For both reasons, we did not pursue *in vivo* experiments at this time. Nevertheless, the processes by which anti-Qβ-coated Vidu is taken up and activates pDCs in the TME would be expected to be like those seen with pDCs in PBMCs, particularly given clinical findings of a strong IFN response induced by intratumoral delivery of Vidu in patients with advanced melanoma (18).

The results presented here suggest that nanoparticles containing TLR9 that are coated with an optimal and controlled concentration of a BDCA2 ligand might be able to activate pDCs without relying on antibody opsonization of the particles. Such agents could have 3 advantages over Vidu. First, targeting a receptor responsible for uptake of immunostimulatory particles by pDCs (BDCA2) that is distinct from the receptor responsible for uptake by other cells (FcγRs) could allow for more specific targeting of pDCs. Second, the BDCA2 ligand and concentration could be controlled, and so optimized based on the window where such targeting activates the pDC without cross linking BDCA2 to the point where activation of the pDC is turned off. Third, such particles could have anti-tumor activity with the first dose, thereby avoiding the need for a priming dose designed to generate anti-Qβ. A variety of molecules that express galactose-terminated glycans are known to be ligands for BDCA2 and could serve to target immunostimulatory particles to intratumoral pDCs. Such targeted particles are currently under evaluation in our laboratory.

## Data Availability

The raw data supporting the conclusions of this article will be made available by the authors, without undue reservation.
