# Supplementary figures and images for: BDCA2 plays a central role in the binding, internalization and response of plasmacytoid dendritic cells to vidutolimod
Source: Front Immunol. 2026 Feb 12;17:1769287. doi: 10.3389/fimmu.2026.1769287 (PMC12951047; doi:10.3389/fimmu.2026.1769287)

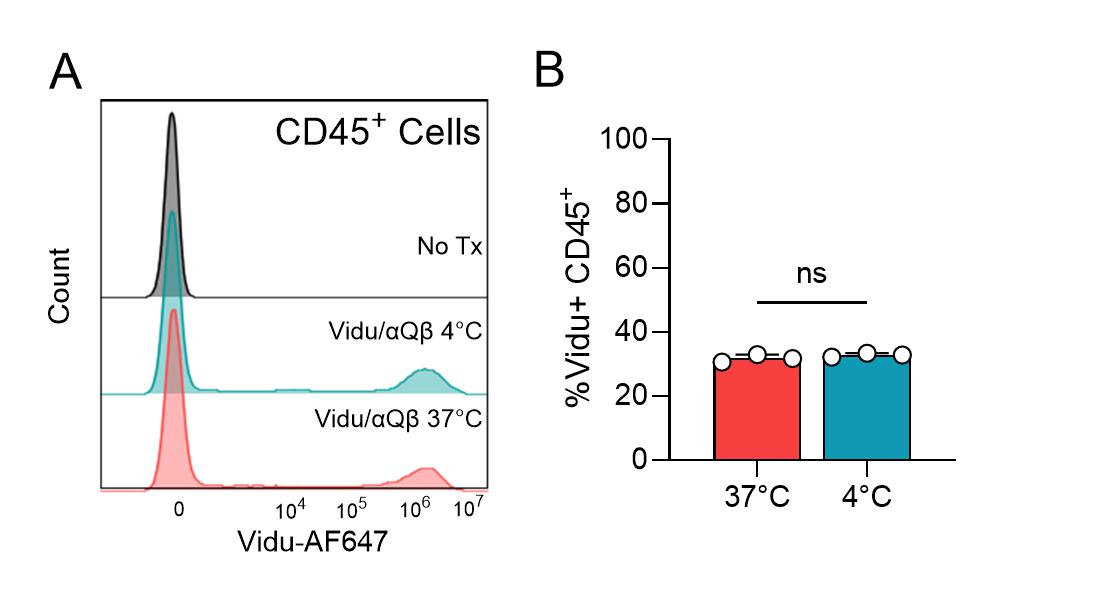

Supplement: Supplementary file 1 [file Image1.jpeg]

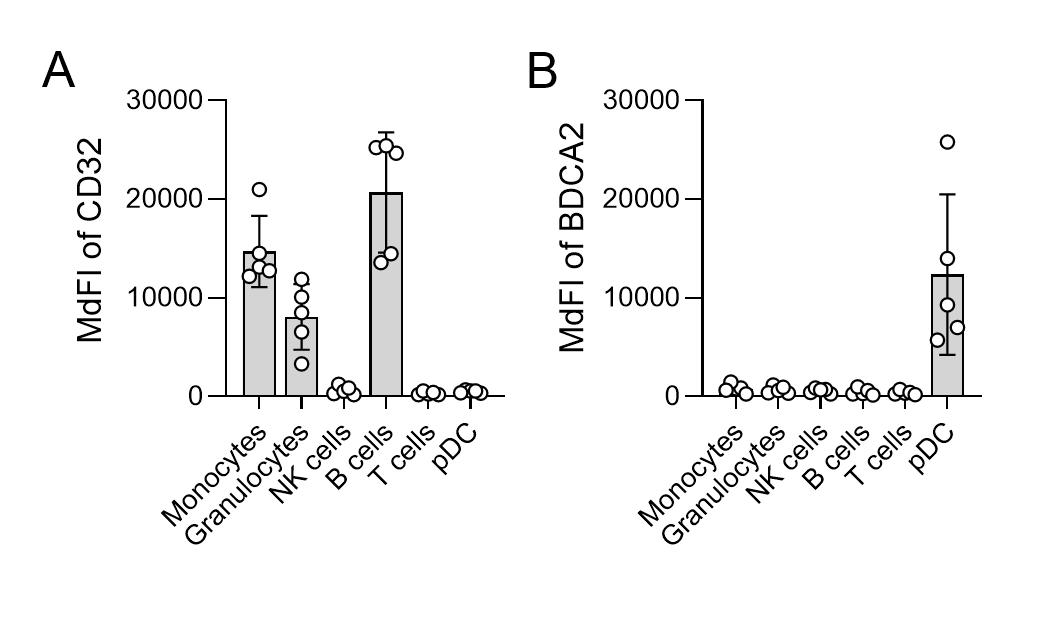

Supplement: Supplementary file 2 [file Image2.jpeg]

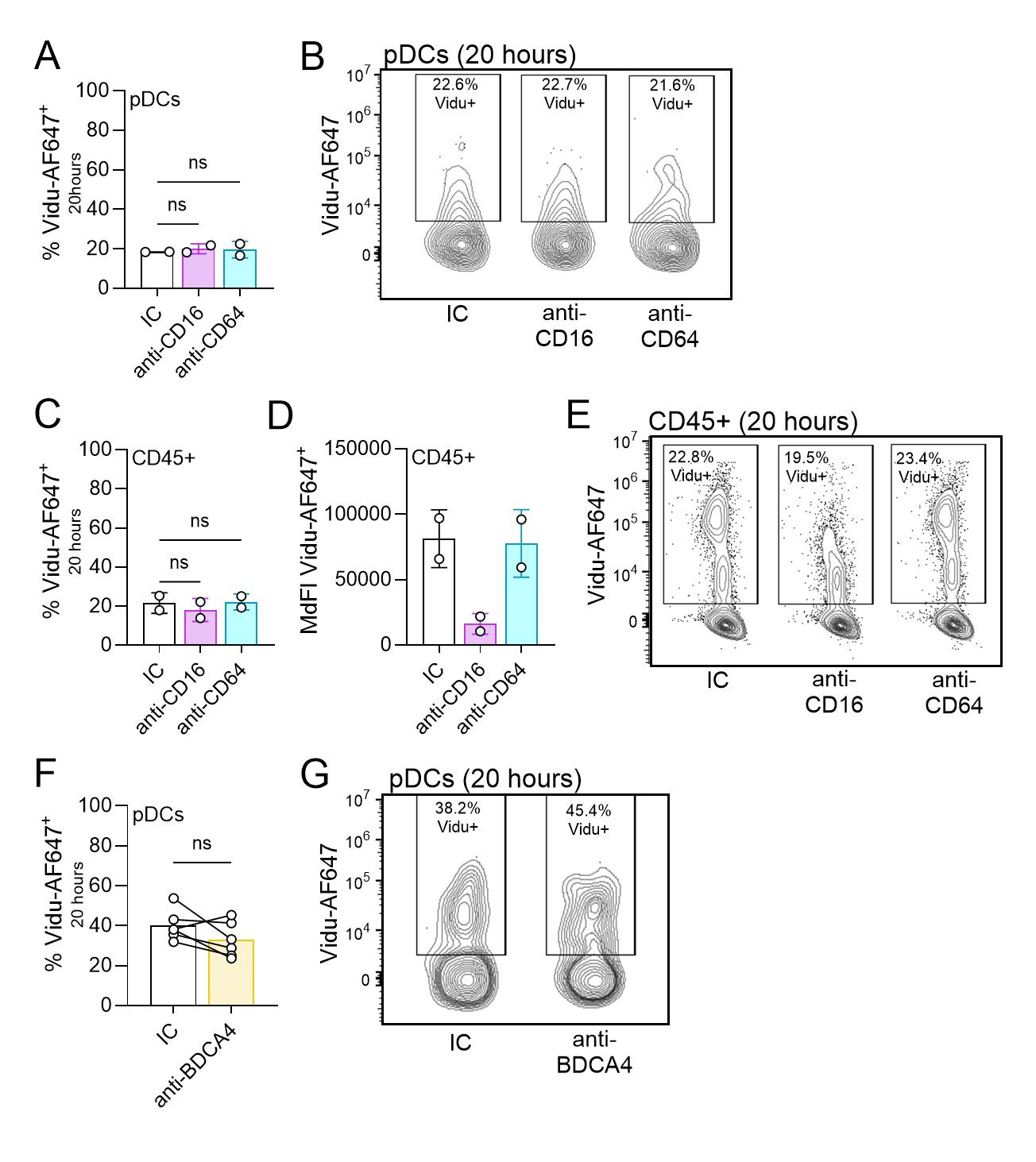

Supplement: Supplementary file 3 [file Image3.jpeg]

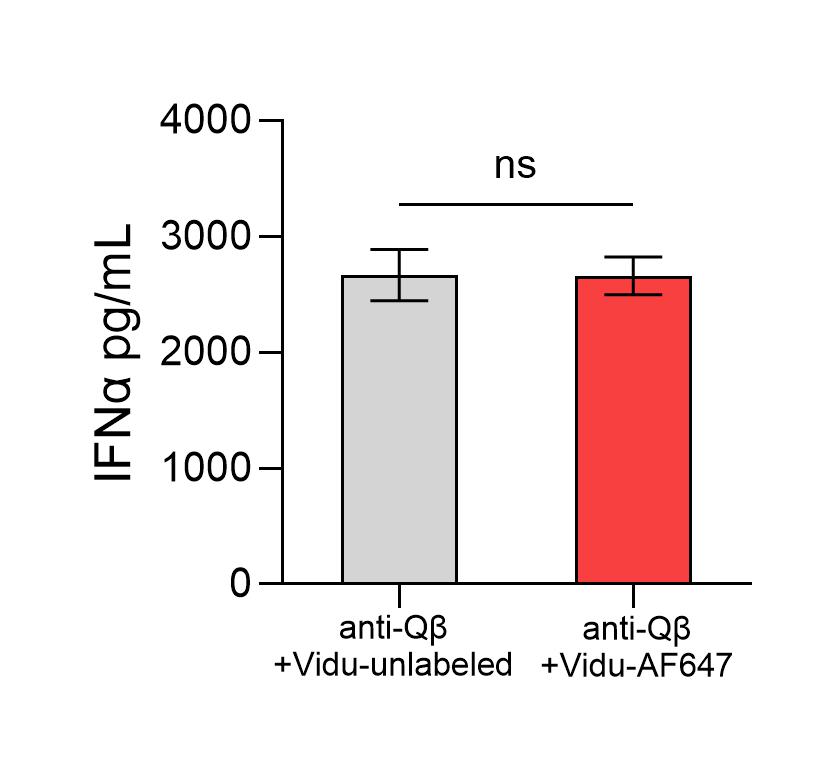

Supplement: Supplementary file 4 [file Image4.jpeg]

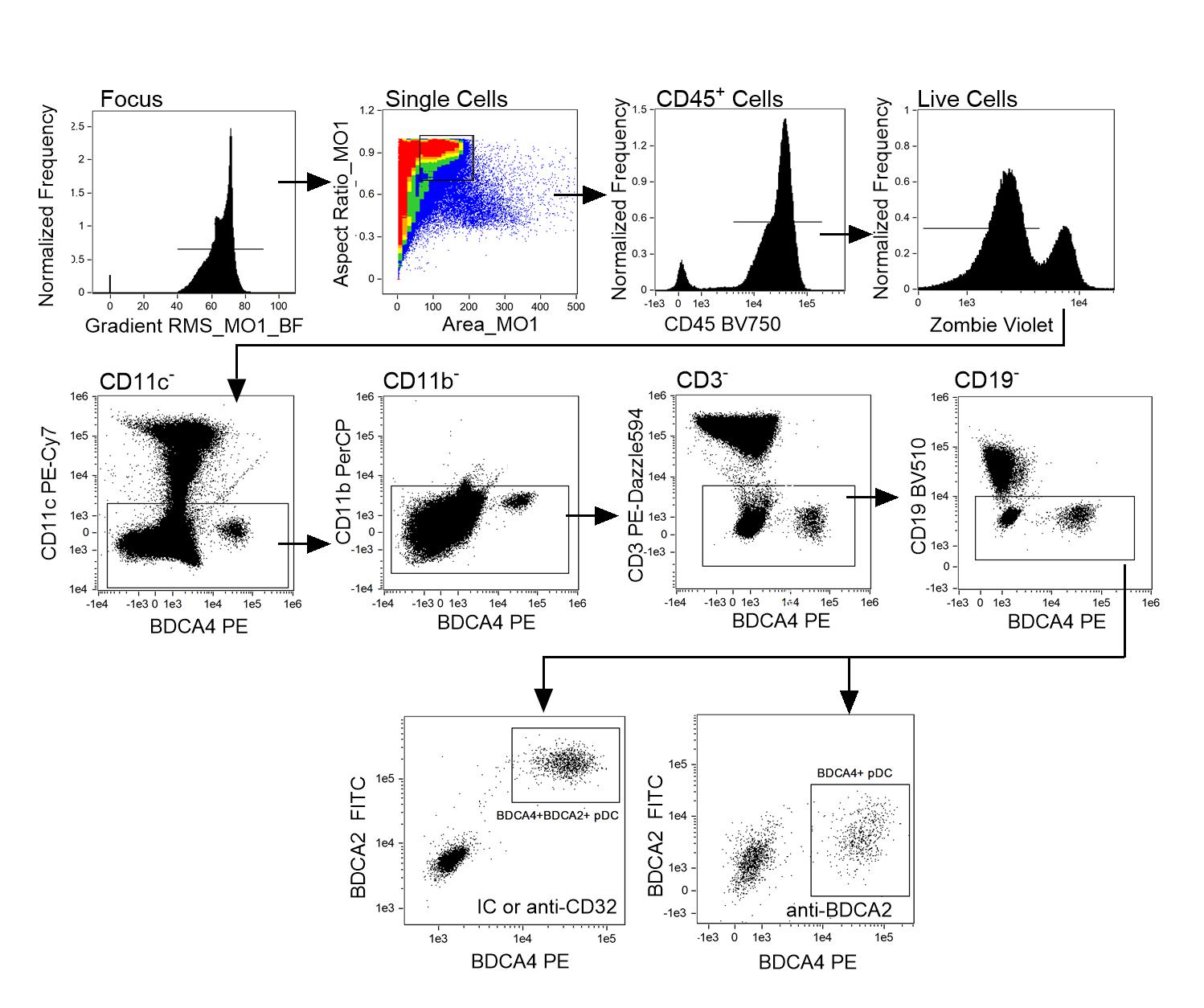

Supplement: Supplementary file 5 [file Image5.jpg]
